# Presumptive Identification of Smooth *Brucella* Strain Antibodies in Canines

**DOI:** 10.3389/fvets.2021.697479

**Published:** 2021-07-08

**Authors:** Alyssa B. Helms, Orsolya Balogh, Rebecca Franklin-Guild, Kevin Lahmers, Clayton C. Caswell, Julie T. Cecere

**Affiliations:** ^1^Department of Small Animal Clinical Sciences, Virginia-Maryland College of Veterinary Medicine, , Blacksburg, VA, United States; ^2^Department of Population Medicine and Diagnostic Sciences, College of Veterinary Medicine, Cornell University, Ithaca, NY, United States; ^3^Department of Biomedical Sciences and Pathobiology, Virginia-Maryland College of Veterinary Medicine, Blacksburg, VA, United States

**Keywords:** brucellosis, canine, abortus, smooth, seroprevalence, antibodies, suis

## Abstract

Brucellosis is a zoonotic disease caused by a Gram-negative coccobacillus. There are four *Brucella* strains of zoonotic importance in our domestic species, subdivided by their culture phenotypes: *Brucella abortus* (*B. abortus*), *B. melitensis, B. suis* (smooth strains) and *B. canis* (rough strain). Dogs can serve as hosts for all four of the zoonotic strains; however, routine serologic testing in dogs has been limited to the identification of *B. canis* antibodies. The aim of our study was to identify smooth *Brucella* strain antibodies in canines. We hypothesize that the *Brucella abortus* Fluorescence Polarization Assay would be successful in identifying smooth Brucella strain antibodies in canines. Ninety-five dogs, including forty-five hog hunting dogs were screened for circulating antibodies to any of the four zoonotic strains of the bacteria utilizing a combination of Canine *Brucella* Slide Agglutination Test (CBSA), *Brucella canis* Agar Gel Immunodiffusion II test (AGIDII), *Brucella abortus* Card Agglutination Test (BCA), and the *Brucella abortus* Fluorescence Polarization Assay (FPA). Test interpretation results yielded a 0% (0/95) smooth *Brucella* strain seropositivity rate, with 2% (2/95) of dogs yielding inconclusive rough *Brucella* strain serology results (0–2% rough strain seropositivity rate). Additionally, a retrospective portion of the study was performed to identify sera containing circulating antibodies to any of the smooth strains of *Brucella* by testing previously banked canine serum samples stored at Cornell's Veterinary Diagnostic Laboratory from 2018 to 2019 via *Brucella abortus* FPA. Of the 769 serum samples tested, 13/769 (1.7%) yielded an inconclusive result, 725/769 (94.2%) were negative, 30/769 (4%) yielded a positive FPA test result, and 1/769 (0.1%) had to be excluded due to insufficient sample remaining to perform the diagnostic test. Of the 30 FPA positive canine serum samples, 97% (29/30) also tested positive on the CBSA test. Additionally, there was a statistically significant (*p* < 0.0001) likelihood of altered (spayed/neutered) and mixed breed dogs to be FPA positive when compared to intact, purebred dogs, respectively.

## Introduction

*Brucella* is a small gram-negative, intracellular, facultative aerobic coccobacillus that is the causative agent of the disease known as brucellosis (1). Brucellosis is a zoonotic disease most well-known for causing reproductive loss in many mammalian species across the globe (1). The highest incidence of disease in humans has been reported in developing countries due to the consumption of raw and unpasteurized animal products (1, 2). In animal hosts, the bacteria is most well-known to be transmitted venereally due to tropism for reproductive tissues. However, *Brucella* has also been documented to be transmitted through mucous membrane contact with bodily secretions such as reproductive fluids, aborted materials and milk predominately, but also urine, respiratory secretions and saliva to a lesser degree (3–5). Following host infection, *Brucella* bacteria become sequestered within phagosomes of the reticuloendothelial system, by evading or suppressing host bactericidal mechanisms and beginning replication within these cells (6–8). *Brucella* especially prefers the cells contained within the reproductive tract, due to the presence of erythritol and fructose which promote bacterial growth (9). In general, clinical signs of infection may include: infertility, abortion, epididymitis, orchitis, diskospondylitis, and undulating fever (10, 11). Additionally, asymptomatic carrier status is also described (12), and infection is often considered to be life-long due to the inability of current antimicrobial strategies to fully clear the organism from the body (13).

There are currently twelve recognized species of *Brucella* that have been identified and named including *Brucella abortus, Brucella suis, Brucella canis, Brucella ovis, Brucella melitensis, Brucella neotomae, Brucella inopinata*; *Brucella microti, Brucella pinnipedialis, Brucella ceti, Brucella vulpis*, and *Brucella papioni* (14). However, the five *Brucella* species that are considered to be of upmost importance in our domestic animal species along with their preferred host include *Brucella abortus* in cattle; *Brucella suis* in pigs and feral swine; *Brucella melitensis* in goats and sheep; *Brucella ovis* in sheep, and *Brucella canis* in dogs (15). Historically, *Brucella* species have been identified to have a distinct host preference, which allowed scientists to name and group the species accordingly. More recently, the understanding of *Brucella*'s host preferences has been debated due to the bacteria's ability to readily adapt and evade host immunity, along with a broad number of case reports indicating much variability in each *Brucella* specie's ability to infect hosts outside of the traditional host-preference classifications.

The causative agent of brucellosis infection in the domestic dog has historically been recognized as a rough strain of the bacteria, *Brucella canis* (16). The seroprevalence of *B. canis* among dogs in the United States is difficult to estimate, and varies depending on the subpopulation of dogs screened with an estimated nation-wide prevalence of ~5.6% (17). Interestingly, recent studies have estimated higher seroprevalence ranges, eluding to a growing concern that this disease could be on the rise in the canine population within the United States. One study conducted between 2007 and 2016 showed *B. canis* prevalence ranged from 0.4% in non-commercial breeding facilities, up to 83% in commercial breeding facilities in the United States (11). Another recent seroprevalence study of dogs rescued from South Dakota Indian reservations from 2015 to 2019 revealed an overall apparent *B. canis* seroprevalence rate of 6.8%, or adjusted true prevalence estimated at 29.4% (18). More recently, canine infection with smooth *Brucella* strains outside of the traditional host range, i.e., *B. suis, B. melitensis* and *B. abortus*, have been evidenced by many case reports (19–25). Smooth strains of *Brucella* are, in general, considered more virulent due to the presence of a non-endotoxic lipopolysaccharide (LPS) coating which allows the bacteria to evade the host's innate immune response (26, 27). Therefore, in general, the smooth *Brucella* species are known to cause more significant disease and carry higher zoonotic potential. Thus, cause for concern has arisen from the scientific community regarding canine infection with the smooth strains of *Brucella*, and the risk of transmission to humans.

Although at present, the United States is considered a “brucellosis free” country, the smooth strains of the disease still exists in several wildlife reservoirs including bison and elk within the Greater Yellowstone Area, and within the feral swine population across the country (28). Canine infection with the smooth strains of *Brucella* has been well-documented in multiple case reports, with the most notable risk factors being: contact with infected livestock or hoof stock (21), contact with infected feral swine (23, 24), and the consumption of infected raw meat and/or unpasteurized dairy products (29, 30). At present, the estimated incidence and prevalence of dogs infected with the smooth strains of *Brucella* is unknown. To the authors' knowledge, only one prevalence study has been conducted on a cohort of 571 Mississippi shelter dogs utilizing the Buffered Acidified Plate Antigen (BAPA) test, in which no serologically positive dogs were identified (31). However, study limitations including geographical limitations, unknown history and therefore unknown risk factors for smooth brucella strain infection, and choice/lack of validation of the serologic (BAPA) test utilized.

Currently, the most commonly utilized serologic tests that have been used to diagnose brucellosis infection in dogs are targeted to only recognize antibodies against the rough canine-specific strain of brucellosis, i.e., *B. canis*, and will not detect antibodies to any of the smooth strains of *Brucella* (i.e., *B*. *suis*, B. *abortus*, or B. *melitensis*) (5). This is due to differences between the LPS cell wall morphologies, with smooth strains possessing an O-antigen side chain on the LPS and rough strains lacking an O-antigen side chain on the LPS (5, 32). It is these differences between cell-wall morphologies that comprise the differences between serologic tests, as these tests evaluate the host's antibody response against *Brucella* cell wall surface antigens (5). Therefore, serologic tests designed to detect smooth *Brucella* species will not cross react with the rough strains of the bacteria (5).

Currently, there is no serologic test validated for the detection of antibodies to the smooth strains of *Brucella* in canines. Rather, previous reports have used a number of different serologic tests that have been validated for the detection of smooth *Brucella* infection in livestock species. A list of some of the different serologic tests utilized include, but are not limited to, the *Brucella* Card Agglutination Test, Rose-Bengal Test, BAPA Test, and Serum Tube Agglutination Test. In livestock species, the prevalence of false positive results with many of these serologic tests requires that all positive serology results be verified with additional follow-up confirmatory testing modalities. The gold standard for the confirmation of brucellosis infection in any species is via positive culture result from either blood or tissue. Unfortunately, false negative culture results are very common, with low and highly variable published sensitivity rates ranging from 10 to 90% (33). Because the bacteria is so fastidious and slow growing, negative culture results can be due to a number of reasons, including: overgrowth of contaminate bacteria, absence of the bacteria in the cultured specimen, and inappropriate culture conditions (34). Recently, the United States Department of Agriculture (USDA) added the Fluorescent Polarization Assay (FPA) as an approved test for the confirmation of brucellosis infection in cattle, bison and swine (35). The FPA test has been repeatedly shown to be an efficient, economical, and highly accurate serum test validated for the confirmatory diagnosis of brucellosis in cattle, swine, sheep, goats, bison, and cervids due to sufficient cross reactivity between the three common smooth *Brucella* strains, i.e., *B. abortus, B. melitensis*, and *B. suis* (36), and has also been utilized to confirm human brucellosis cases due to zoonotic infection for years. Therefore, given the FPA test's successful validation across many different species and common smooth *Brucella* strain types, it is plausible to consider that this test could also be successful in detecting smooth strain *Brucella* infection in canine sera.

There is much debate on the validity and reliability of serologic tests in the diagnosis of the smooth strains of brucellosis in canines, as there is a significant need for validation studies in canines. Clinically, many veterinarians utilize the commercially available *Brucella abortus* Card Agglutination Test (BCA) to aid in the diagnosis of smooth *Brucella* stain infection in canine cases where there is a high index of suspicion. However, limited research has been conducted to support the decision to utilize this test. Additionally, although feral swine hunting dogs are considered to be at increased risk of smooth *Brucella* strain infection (23), it is unknown how common infection is within this population in the United States. With these facts in mind, our study was divided into two parts, prospective and retrospective. The goal of the prospective aspect of our study was 2-fold. First, to detect antibodies to the smooth strains of *Brucella* in sera of clinically healthy dogs with (i.e., feral swine hunting dogs) and without (i.e., dogs presenting for routine spay/neuter) known risk factors for infection. Second, as no agreement exists on which serologic test should be utilized for the diagnosis of the smooth strains of brucellosis in canines, we chose to utilize and report the results of the commonly used BCA test as well as the broadly validated confirmatory FPA tests for comparison. Lastly, the goal of the retrospective aspect of this study was to detect antibodies and evaluate the prevalence of seropositivity to smooth *Brucella* strains via FPA testing of banked canine sera from dogs that were previously screened for *Brucella canis*. In doing so, our hope was to shed light on if and how the veterinary community should be screening dogs for smooth *Brucella* strain infection. Particularly dogs with clinical evidence of brucellosis infection, an unknown background, or known risk factors for smooth *Brucella* strain infection.

## Materials and Methods

### Prospective Study Samples

Ninety-five dogs of various ages (12 weeks to 9 years of age), sex (males *n* = 57, females *n* = 38), spay/neuter status, parities and breeds were included in this part of the study. All animals were apparently healthy at the time of sampling with inclusion criteria being limited to facility accessibility and owner consent. Reporting of active feral swine hunting history was determined via self-reporting by the owner. Blood samples were collected from all dogs using standard venipuncture protocols approved by the Virginia Tech Institutional Animal Care and Use Committee (IACUC Protocol Number: 18-220). The blood was placed into a sterile, 5 mL serum tube and allowed to clot at room temperature for 30 min before centrifugation at 1500 g for 10–15 min. After centrifugation, the serum was removed, transferred to a sterile 5 mL red top tube, and stored in a freezer at −20°C until analysis.

Serologic testing was performed at Animal Health Diagnostic Center at the Cornell University College of Veterinary Medicine, which is the reference laboratory for canine brucellosis testing. Testing for *Brucella canis* included the Canine *Brucella* Slide Agglutination Test (CBSA) and *Brucella canis* Agar Gel Immunodiffusion II (AGIDII). The CBSA is performed using an in-house produced killed whole-cell antigen made from an M- strain of *B. canis* stained with Rose Bengal. Equal volumes of each patient serum and 0.2M 2-Mercaptoethanol (2-ME) are mixed together on an agglutination plate and allowed to sit for 30 s. The slide antigen is then mixed in with each treated sample and rocked for 3 min before being evaluated on an inverted microscope at 4X for graded levels of agglutination and clearing of the antigen. Samples that exhibit 3-4+ agglutination are considered positive and 0-2+ agglutination are considered negative. The AGIDII is performed using an in-house prepared cytoplasmic antigen made from an M- strain of *B. canis* and using an AGID agar prepared at the AHDC Media Processing Center. A template is used to punch a seven-well pattern (a center well with six surrounding wells) in the AGID agar. Antigen is loaded into the center well and the outer wells are loaded with equal volumes of patient serum and positive control in alternating fashion. The plate is incubated in a humid box at 25–29°C in ambient air for 18–24 h on a level surface. After incubation, precipitin lines are observed using an illuminating device. Lines of identity or weak positive reactions between the sample well and the antigen well are determined to be AGIDII Positive. Samples that form no precipitin lines are called AGIDII Negative. Lines of non-identity are reported as AGIDII Suspect and samples exhibiting lines of partial identity may be reported as AGIDII Positive (if CBSA positive) or AGIDII Suspect (if CBSA negative). Final results are interpreted in conjunction with the CBSA as either Positive, Negative or Inconclusive. Controls using well-characterized positive stock sera and a negative serum pool are used in both of these tests on every testing day to confirm the test is working properly.

For detecting antibodies against smooth strains of *Brucella*, the *Brucella abortus* Card Agglutination Test (BCA) and the *Brucella abortus* Fluorescence Polarization Assay (FPA) were run concurrently. The BCA is performed using *B. abortus* buffered *Brucella* antigen prepared at the National Veterinary Services Laboratory (NVSL) in Ames, Iowa and Becton Dickinson Brucellosis Testing Cards. The patient serum and *Brucella* antigen are each loaded onto the marked teardrop-shaped well in 30 μl volumes. The serum and antigen are mixed together using a stirrer and then the card is placed on a rocker for 4 min. Reactions are observed promptly at the end of the rocking period using an illuminating device. Samples showing no characteristic clumping and having a grainy appearance with dispersal of particles are considered negative. Samples showing characteristic macroscopic agglutination with moderate to large clumps are considered positive. Some species other than bovine (such as cervids, equine, porcine, and canine) will often exhibit a different clumping due to non-specific cross-reaction. All BCA positive samples are then run on FPA tube for confirmation. Positive and negative controls from NVSL are run alongside patient samples on every testing day to ensure the test is performing properly. The FPA is a test that uses an O-polysaccharide (OPS) extracted from *B. abortus* and labeled with fluorescein. The fluorescence polarization instrument is used to measure the polarization state of the light emitted by the OPS conjugate and reports the values in millipolarization units (mP). When no antibodies are present, the polarization is low. Polarization increases when antibodies bind to the conjugate. The FPA test is performed either in microwells (strips) as a screening test or in tubes as a confirmation test using the FPA test kit and instrumentation purchased from Ellie Lab. The FPA microwell test is run with the Sentry ® 2000S ™ strip reader and 8-well strips. Twenty microliter of the patient sample is pipetted into a well followed by 180 μl of reaction buffer. This strip is loaded into the machine where the wells are agitated and incubated for 2 min and then the mP is read. 10 μl of conjugate is added to each well with the machine agitating the wells again, incubating for an additional 2 min and the mP is read a second time. The machine calculates the delta mP for all controls (three negatives and one positive) and each sample, comparing the results of the sample to the controls to determine the final result. Samples with a delta mP value <10 above the mean negative control value are considered negative. Samples with a delta mP value ≥ 10 above the mean negative control value must be retested in duplicate for confirmation using the FPA tube test. For the FPA tube test, 20 μl of patient sample is pipetted into each of two test tubes followed by 1 ml of reaction buffer and vortexed gently to mix. The tubes are incubated at room temperature for 3–30 min and read in a Sentry ® 200 ™ tube reader. Ten microliter of conjugate is added to each tube and vortexed gently to mix. The tubes are incubated again at room temperature for 2–5 min and read a second time in the tube reader, with the machine calculating the delta mP values. Samples with both tests having a delta mP value of <10 above the mean negative control value are considered negative. If one or both tests have a delta mP value of 10–20, the sample is considered suspect. If both tests have a delta mP value >20, the sample is considered positive.

Additionally, for participating dogs that were presenting for routine spay and neuter, discarded epididymal and uterine tissue samples were sterilely collected, sectioned and stored in a freezer at −80°C following acquisition. Alternatively, when available, intact male dogs were manually ejaculated to obtain a semen and prostatic sample into a sterile bag, and vaginal swabs were collected from intact female dogs by passing a sterile, guarded Kalayjian swab into the vagina. Both semen samples and vaginal swabs were stored in a freezer at −80°C following acquisition. Tissue, sperm or vaginal swab samples from dogs that tested serologically positive on *Brucella canis* serology or the *Brucella abortus* FPA were transferred to a BSL3 lab and subjected to culture. Clinical samples were routinely grown in brucella broth (BD, Sparks, MD) at 37°C with constant shaking or on Schaedler blood agar (SBA), composed of Schaedler agar (Acumedia, Burton, MI) containing 5% defibrinated bovine blood (Quad Five, Ryegate, MT) at 37°C with 5% CO_2_.

### Retrospective Study Samples

**Figure 1:**
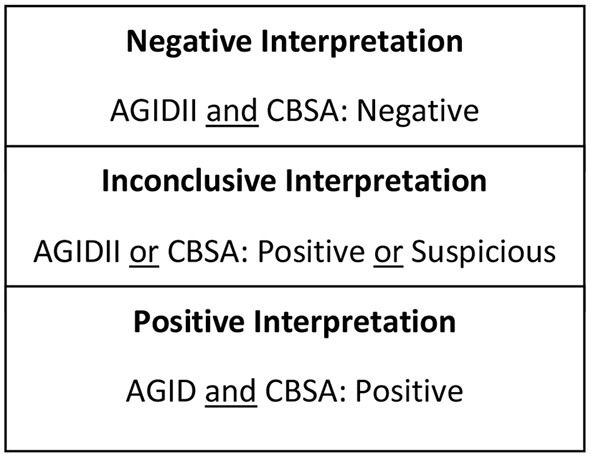
Test interpretation utilized for serologic screening for the rough strain of brucellosis (*Brucella canis*).

**Figure 2:**
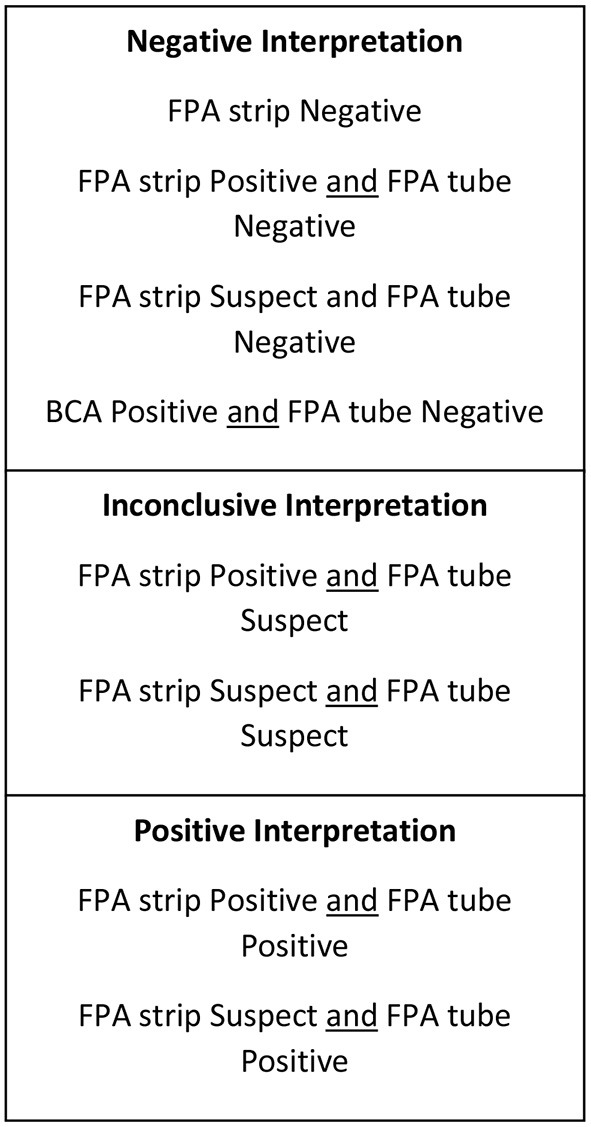
Test interpretation utilized for serologic screening for the smooth strains of brucellosis (*Brucella abortus, suis, melitensis*).

Seven hundred and sixty-nine serum samples, frozen and stored at −20°C, submitted to the Animal Health Diagnostic Center at the Cornell University College of Veterinary Medicine for *Brucella canis* screening between 2018 and 2019 were subjected to antibody testing for smooth strains of *Brucella* via *Brucella abortus* Fluorescence Polarization Assay (FPA). All frozen-thawed serum samples were from canines of various ages, sex, spay/neuter status, and geographical locations that had been previously screened for *Brucella canis* via CBSA and AGIDII. Inclusion criteria included a test interpretation result of either “positive” or “inconclusive” following CBSA and AGIDII screening (Figure 1). Serum samples were initially tested by *Brucella* FPA microtiter strip test, which utilizes a small test volume in a format which allows for the testing of eight samples at a time. Results are measured in milipolarization units (mP) above the mean negative control value of 10–20, which is referred to as the delta mP. Any *Brucella* FPA microtiter strip test interpreted as “suspect” (delta mP of 10–20) or “positive” (delta mP > 20) was retested by FPA tube in duplicate. The tube tests allow for a higher dilution of the serum, thereby decreasing components that can cause a non-specific polarization reaction. The FPA tube results were considered “negative” if both tubes had a delta mP of <10, “suspect” if either tube has a delta mP of 10–20 and “positive” if both tubes have a delta mP of >20. All FPA microtiter strip test “negative” samples were considered truly negative, and did not undergo FPA tube testing. FPA results on all canine samples were reported with a disclaimer as the test is not validated for this species. Finalized FPA interpretations for the combined strip and tube test results are listed in Figure 2.

### Test Interpretation

Test interpretation for *Brucella canis* serology (AGIDII and CBSA) results were determined by the Animal Health Diagnostic Center at the Cornell University College of Veterinary Medicine, and are listed in Figure 1. Suggested test interpretation for the serologic tests (BCA and FPA tests) utilized for the detection of smooth *Brucella* strain antibodies were determined by expert opinion, and are outlined in Figure 2. It is important to reiterate that the test interpretation utilized for the FPA and BCA tests in this study are based on a culmination of current knowledge, expert opinion, clinical suspicion, and interpretation utilized by previous case reports, as currently no validated serologic test for the smooth strains of brucellosis exists for canines.

### Data Analysis

For both prospective and retrospective data, all data was entered and maintained in an electronic spreadsheet, which was then used for statistical analysis. Animal gender, breed, and geographic location were determined by the reporting veterinary practitioner, reference laboratory, or labeled as “not specified” (Nos) when descriptive data was not provided. Associations between categorical variables were assessed using Fisher's exact tests. A *p*-value of < 0.05 was utilized to determine statistical significance. All analyses were performed using SAS version 9.4 (Cary, NC, USA). When analyzing results based on breed or sex, samples where descriptive data was not provided (i.e., Nos) were excluded from statistical analysis.

## Results

### Prospective Study Samples

Of the 95 samples collected, seventy-six were from client owned dogs residing in the following states: Tennessee, Louisiana, Kentucky, South Carolina, Massachusetts, Texas, New York, Florida, Georgia and Virginia. Forty-five of the client owned dogs were classified as active feral swine hunting dogs. Additionally, nineteen shelter owned dogs were enrolled from shelters in Georgia, Florida, and Virginia. Serologic testing for *Brucella canis* resulted in 98% negative samples (93/95) and 2% inconclusive samples (2/95) (Table 1). Of the inconclusive results, one test result (a male, intact, shelter dog from Florida) was “suspicious” on AGIDII and the other (a male, intact, feral swine hunting dog from Tennessee) was “positive” on CBSA but negative on AGIDII. Three culture attempts were made on the epididymal tissue of the shelter dog from Florida which yielded contaminant growth only. Test results for the smooth strains of brucellosis, including the BCA and the FPA tests, for all 95 samples are reported in Table 2. The overall interpretation of the smooth strain serology results was 0% positive, with 38/95 (40%) BCA tests yielding a positive result, and 0/95 (0%) FPA tests yielding a positive result (Table 2).

**Table 1:** Test results and interpretation of rough strain *Brucella canis* tests for 95 prospective samples.

**Test result**	**AGIDII**	**CBSA**	**Interpretation**	**% Samples**
Positive	0	1	0	0
Negative	94	94	0	97
Inconclusive/ Suspect	1	0	2	2

**Table 2:** Test results and suggested interpretation of smooth strain *Brucella* tests for 95 prospective samples.

**Test result**	**BCA**	**FPA strip**	**Suggested**	**% Samples**
			**interpretation**	
Positive	38	0	0	0
Negative	57	95	95	100
Inconclusive/Suspect	N/A	0	0	0

### Retrospective Study Samples

Of the 769 canine serum samples tested, most (*n* = 424, 55%) were from intact animals (33%, *n* = 253 females; and 22%, *n* = 171 males) with altered animals (*n* = 300, 39%) encompassing both spayed females (*n* = 154, 20%) and castrated males (*n* = 146, 19%). For 45 samples (6%), the sex of the animal was not specified. Samples were submitted from many different referral laboratories and referral veterinarians across the United States, including the following states: Alabama (2), Arkansas (79), Arizona (2), California (138), Colorado (31), Connecticut (1), Florida (8), Iowa (13), Idaho (1), Illinois (IL), Indiana (1), Kentucky (4), Louisiana (61), Massachusetts (50), Maryland (4), Maine (2), Michigan (7), Minnesota (8), Missouri (1), Mississippi (5), Montana (6), North Carolina (7), North Dakota (1), New Hampshire (5), New Mexico (4), New York (148), Ohio (2), Oklahoma (9), Oregon (4), Pennsylvania (9), Rhode Island (1), Tennessee (34), Texas (19), Virginia (6), Washington (13), Wisconsin (15). Additionally, serum samples from Ontario, Canada (*n* = 42), Prince Edward Island, Canada (*n* = 2) and Brazil (*n* = 17) were utilized. Regarding breed, the majority (*n* = 502, 65.3%) were reported as an American Kennel Club recognized purebred canine, with mixed breed (*n* = 156, 20.3%), and breed not specified (*n* = 111, 14.4%) also reported.

*Brucella* serology results for *Brucella canis* (including CBSA and AGIDII) screening for the 769 samples were as follows: 1% (4/769) yielded invalid test results, 63% (489/769) yielded a positive result, and 36% (276/769) yielded an inconclusive result (Table 3). Test results for the smooth strains of brucellosis utilizing FPA are reported in Table 4, including suggested test interpretations. Of the 769 sera tested, 13/769 (1.7%) yielded an inconclusive result, 725/769 (94.2%) were negative, 30/769 (4%) yielded a positive FPA test result, and 1/769 (0.1%) yielded an invalid test result (Table 4). Invalid test results were due to not enough sample remaining to perform the diagnostic test.

**Table 3:** Test results and interpretation of rough strain *Brucella canis* tests for 769 retrospective samples.

**Test result**	**AGIDII**	**CBSA**	**Interpretation**	**% Samples**
Positive	489	619	489	63
Negative	81	146	0	0
Inconclusive/ Suspect	195	0	276	36
Invalid	4	4	4	1

**Table 4:** Test results and suggested interpretation of smooth strain *Brucella* tests for 769 retrospective samples.

**Test result**	**FPA strip**	**FPA tube**	**Suggested**	**% Samples**
			**interpretation**	
Positive	138	30	30	4
Negative	594	131	725	94.2
Inconclusive/Suspect	36	13	13	1.7
Invalid	1	0	1	0.1

Statistical analysis regarding categorical variables of smooth strain FPA interpretation results were as follows. No statistically significant pattern was identified between the outcome of the FPA interpretation and the AGIDII result (*p* = 0.3). However, there was a statistically significant pattern (*p* = 0.029) between the outcome of the FPA interpretation and the CBSA result, with 97% (29/30) of FPA interpretation “positive” samples also testing positive on the CBSA test. Serum from both spayed and castrated gender classifications were more likely (*p*-value: < 0.0003) to be positive on FPA test interpretation than the intact dogs (Table 5). A significant association was also identified between breed and outcome of the FPA test interpretation, with mixed breed dogs being more likely to have a positive FPA test interpretation result than purebred dogs (*p* = < 0.0001) (Table 6). Forty-one and one-hundred and eleven samples were excluded due to the absence of reported patient gender and breed, respectively. Geographic information regarding the locality of the 30 FPA test interpretation “positive” samples are listed in Table 7.

**Table 5:** Breakdown of smooth strain interpretation by patient gender (retrospective samples).

	**Smooth** ***Brucella*** **strain interpretation**			
**Gender**	**Inconclusive**	**Negative**	**Positive**	**Total**
Castrated male	3	129	13	145
Spayed female	3	139	12	154
Intact female	3	249	1	253
Intact male	3	164	4	171
Total	12	681	30	723

*Samples with gender not specified were excluded from the analysis*.

**Table 6:** Breakdown of smooth strain interpretation by breed (retrospective samples).

	**Smooth** ***Brucella*** **strain interpretation**			
**Breed**	**Inconclusive**	**Negative**	**Positive**	**Total**
Mixed Breed	2	137	17	156
Purebred	5	487	10	502
Total	7	624	27	658

*Samples with breed (purebred or mixed breed) not specified were excluded from the analysis*.

**Table 7:** Geographic location of the 30 FPA test interpretation “positive” samples.

**State**	**Number of FPA “positive” results**
Arkansas	1
California	7
Colorado	2
Idaho	1
Massachusetts	4
Mississippi	1
North Carolina	1
New Hampshire	1
New York	3
Tennessee	4
Texas	2
Wisconsin	3

Of the 763 *Brucella canis* serology interpretation results, no statistically significant pattern was identified between *Brucella canis* serology interpretation by gender or breed.

## Discussion

Brucellosis is a calamitous bacterial disease that is considered under-recognized by the CDC in canines (5). The bacteria carries high zoonotic potential as it is readily transmitted through ingestion, inhalation, and mucous membrane contact with milk and reproductive secretions, as well as saliva, feces, urine, raw meat and respiratory secretions to a lesser degree (3–5, 37). Once infected, the disease is associated with significant long term and often life-threatening health implications in both dogs and humans alike, as therapy is often ineffective at clearing the organism completely (13). Although much effort has been given to the control of the disease within the United States livestock population, the surveillance and control of brucellosis infection in canines and the potential for more widespread human infection with the disease due to close contact with canine companions has been vastly overlooked.

Although brucellosis in dogs remains endemic in many parts of the world, including some breeding facilities within the United States, there are currently no mandatory testing regulations (nor regulatory guidelines) for dogs prior to interstate travel, international travel, or adoption (5, 11). At present, the only control strategy regularly practiced within the veterinary community is *Brucella canis* screening of breeding bitches and stud dogs prior to mating, due to the well-known risk of venereal transmission. Although less well-recognized, brucellosis infection within the spayed and neutered canine population has been increasingly recognized, with a recent abstract published by Cheong et al. revealing a proportionally higher number of spayed (33%) and castrated (28%) dogs testing positive on serology for *Brucella canis* compared to intact males (8%) and females (9%) (38). Thus, it is possible that the current exclusive strategy of surveillance testing breeding dogs prior to mating may be vastly under-estimating the current seroprevalence of brucellosis within this canine population in the United States.

In addition to a growing awareness of the disease within the castrated dog population, an increasing number of reports have been published regarding the expanding concern of smooth *Brucella* strain (*B. abortus, B. suis, and B. melitensis*) infections in dogs, especially within the feral swine hunting canine population. Surprisingly, the prospective portion of our study failed to detect any positive smooth *Brucella* strain interpretation results within the feral swine hunting dogs that were sampled (seroprevalence reported at 0%). This could be due to study limitations including small sample size, lack of test validation in canines, and geographic sampling limitations. Therefore, the overall prevalence of smooth Brucella strain infection (i.e., *B. suis*) in feral swine hunting dogs remains ultimately unknown, as a much larger study population would be needed to better estimate true seroprevalence.

Interestingly, in agreement with Cheong et al. (38) our study identified that mixed breed and spayed/neutered canines were much more likely to be interpreted as positive on FPA for smooth *Brucella* strain antibody detection than intact or purebred canines. Unfortunately, due to the retrospective study design, previously reported patient history was limited. Therefore, other potential risk factors which have been previously identified in the literature such as contact with infected livestock or hoof stock (21), contact with infected feral swine (23, 24), the consumption of infected raw meat and/or unpasteurized dairy products (29, 30), importation from a *Brucella* endemic country, or the presence of clinical signs related to brucellosis could not be assessed. Nonetheless, our results suggest that *Brucella* screening recommendations should not be limited to intact animals prior to breeding, and should instead include any canine that has a history of livestock or feral swine exposure, importation from a *Brucella* endemic country, and/or a history of consuming raw meat (especially raw wild game) diets.

All samples in the retrospective portion of our study tested either positive or suspicious on CBSA or AGIDII for *B. canis*, which was the prerequisite for inclusion. Interestingly, we found a significant association between canine serum that was interpreted as positive on the FPA test also tested positive on CBSA. It is the authors' opinion that this finding is probably largely due to observed cross-reaction on the CBSA test. Cross reactivity as evidenced by non-specific agglutination is a common finding with CBSA testing that inherently lowers the test's specificity for *Brucella canis* when interpreted alone. This is why the CBSA test is used and interpreted jointly with the AGIDII test for the serologic diagnosis of *Brucella canis*. Therefore, our findings suggest that for clinical cases with known risk factors or a high index of suspicion for clinical disease, dogs testing positive on CBSA and negative AGIDII test should undergo follow-up testing for a smooth strain of brucellosis.

In regards to the debate of which serologic test should be utilized for the detection of canine antibodies to the smooth strains of *Brucella* (*B. abortus, B. suis*, and *B. melitensis*), there is still a considerable amount of research that needs to be conducted, including validation studies. Our finding of 40% BCA positive and 0% FPA positive samples in the prospective portion of the study suggest that the *Brucella abortus* card agglutination test alone should not be utilized for the diagnosis of smooth *Brucella* strain infection in the canine. Given the high number of positive BCA results noted within a clinically healthy population of dogs, the lack of accompanying FPA positivity, and the low specificity of agglutination tests due to non-specific agglutination, the BCA positive results from this study were considered false positive results. Rather, future research efforts should likely focus on the validation of the *Brucella abortus* Fluorescent Polarization Assay for the diagnosis of smooth *Brucella* strain infection in the canines.

In conclusion, our study supports the finding that brucellosis infection is not exclusive to the intact canine breeding population, and serves as a reminder that spayed and neutered dogs are also at risk of harboring and spreading this zoonotic disease. Additionally, spayed and neutered mixed breed dogs may be more likely to harbor smooth *Brucella* strain infection than the intact and purebred canine populations. Though speculative, we hypothesize that this could be explained by the recent influx in the importation of canines into the United States from *Brucella* endemic countries (39). There is significant need for future research efforts involving the validation of a serologic test for the detection of smooth *Brucella* strain infection in canines. Our results suggest that research efforts should likely focus on validating the *Brucella abortus* Flourescent Polarization Assay, and until further research is conducted, the *Brucella abortus* card agglutination test should not be used as a diagnostic tool in canines. Lastly, once a validated test is made available, regulatory testing recommendations to include both rough and smooth *Brucella* strain serologic testing should be instituted. This should include both intact and spayed/neutered canines with known risk factors for infection including: breeding, intimate contact with livestock or hoof stock, contact with feral swine, the consumption of unpasteurized dairy products, the consumption of raw wild game meat, and importation from a *Brucella* endemic country.

## Data Availability Statement

The original contributions presented in the study are included in the article/supplementary material, further inquiries can be directed to the corresponding authors.

## Ethics Statement

The animal study was reviewed and approved by the Virginia Tech Institutional Animal Care and Use Committee. Written informed consent was obtained from the owners for the participation of their animals in this study.

## Author Contributions

AH, CC, KL, and JC contributed to conception and design of the study. AH and RF-G organized the data. AH wrote the first draft of the manuscript. AH, JC, OB, and RF-G wrote sections of the manuscript. All authors contributed to manuscript revision, read, and approved the submitted version.

## Conflict of Interest

The authors declare that the research was conducted in the absence of any commercial or financial relationships that could be construed as a potential conflict of interest.
